# Botulinum Neurotoxin for Pain Management: Insights from Animal Models

**DOI:** 10.3390/toxins2122890

**Published:** 2010-12-21

**Authors:** Flaminia Pavone, Siro Luvisetto

**Affiliations:** CNR, Institute of Neuroscience-Roma, via del Fosso di Fiorano 64, I-00143 Roma, Italy; Email: siro.luvisetto@cnr.it

**Keywords:** botulinum toxin, SNARE, pain, animal model, analgesia, inflammatory pain, chronic pain, peripheral sensitization, central sensitization, retrograde axonal transport

## Abstract

The action of botulinum neurotoxins (BoNTs) at the neuromuscular junction has been extensively investigated and knowledge gained in this field laid the foundation for the use of BoNTs in human pathologies characterized by excessive muscle contractions. Although much more is known about the action of BoNTs on the peripheral system, growing evidence has demonstrated several effects also at the central level. Pain conditions, with special regard to neuropathic and intractable pain, are some of the pathological states that have been recently treated with BoNTs with beneficial effects. The knowledge of the action and potentiality of BoNTs utilization against pain, with emphasis for its possible use in modulation and alleviation of chronic pain, still represents an outstanding challenge for experimental research. This review highlights recent findings on the effects of BoNTs in animal pain models.

## 1. Historical Overview: From Sausages to Universal Drug

Botulinum neurotoxins (BoNTs) are produced by anaerobic bacteria of the genus *Clostridium* and are the most poisonous biological substances known [1,2]. BoNTs are responsible for botulism, a neuroparalytic syndrome characterized by flaccid paralysis, originating in the peripheral nervous system, and by dysfunctions of the autonomic nervous system [3,4,5,6]. In food borne botulism, the most common form of botulism, BoNTs are ingested with food in which spores have germinated and the organism has grown. Food-borne botulism has accompanied mankind since the beginning (see [7] for extensive review). At the end of the 18th century, some well-documented outbreaks of sausage poisoning in Württemberg, Southern Germany, prompted early systematic botulinum toxin research. Between 1817 and 1822, the German poet and district medical officer Justinus Kerner published the first accurate and complete description of the symptoms of food-borne botulism. He did not succeed in defining the suspected biological poison that he called “sausage poison” or “fatty poison”; however, he developed the idea of a possible therapeutic use of the toxin. In 1895, Emile Pierre van Ermengem, Professor of bacteriology at the University of Ghent, following a botulism outbreak after a funeral dinner with smoked ham in the small Belgian village of Ellezelles, discovered the pathogen anaerobic gram positive bacterium *Clostridium botulinum*. The bacterium was so called because of its pathological association with the sausages (Latin word for sausage = botulus). 

It was not until the middle of 20th century that BoNTs were used as therapeutic drugs in medicine. Research began when Dr. Edward J. Schantz and his colleagues were able to purify botulinum toxin serotype A (BoNT/A) into crystalline form. In 1953, the physiologist Dr. Vernon Brooks discovered that the injection of small amounts of BoNT/A into a hyperactive muscle blocked the release of acetylcholine (ACh) from motor nerve endings, causing temporary “relaxation”. In the 1960s, the ophthalmologist Dr. Alan B. Scott started treating monkeys with BoNT/A, theorizing that its muscle-relaxing effects might help in the treatment of crossed eyes (or strabismus). In the 1980s, Alan Scott introduced the use of BoNT/A as an alternative to conventional surgery to treat human strabismus. After these pioneering studies, therapeutic applications of BoNT/A were extended to a wide variety of neurological disorders associated to spasmodic muscle contractions, for example, the spasmodic torticollis, blefarospams, facial emispams, and dystonia [8]. Nowadays, the clinical indications for BoNT/A are constantly growing, ranging from treatment of overactive skeletal and smooth muscles, to management of hypersecretory and painful disorders such as migraine, trigeminal neuralgia and the myofascial pain syndrome [9,10,11]. 

This review is focused on studies in which BoNTs, mainly BoNT/A, were used as pharmacological treatment against pain in animal models. 

## 2. Cellular Mechanism: General Considerations and Different BoNTs Serotypes

From the plethora of toxins produced by *Clostridium botulinum*, only toxins whose action is restricted to the nervous system, such as BoNTs, are strictly defined as neurotoxins. Other toxins, such as botulinolisin, toxin-C2, exoenzyme-C3, which target different type of cells, mostly non-neuronal, are not included in the BoNTs family. Seven different serotypes of BoNTs have so far been characterized (A-G), and these serotypes are active on many different types of vertebrates [3,4]. BoNTs are secreted as multimolecular complexes together with non toxic accessory proteins (emagglutinins) that do not contribute to the paralyzing effects of BoNTs but protect them from the passage through the acidic and proteolytic environment of the stomach after the ingestion of spore-contaminated food [12,13]. 

BoNTs are proteins of about 1,300 amino acids and consist of three domains of similar size (50 kDa). The NH_2_-terminal domain, which is named L-chain (L = light) domain, is a zinc endopeptidase that represents the catalytic domain expressing the protease activity. The other two domains, which are covalently bound to form the H-chain (H = heavy), are the central domain, responsible for the membrane translocation of the L-chain into the neuronal cytosol, and the COOH-terminal domain, which consists of two equally sized subdomains, responsible for the neurospecific binding [14,15,16].

The cellular action of BoNTs occurs as a four-step mechanism: (i) binding of BoNTs on the neuronal presynaptic membrane, via interaction with gangliosides, synaptic vesicle protein 2 and/or synaptotagmin, depending on the serotype [16,17,18]; (ii) internalization of BoNTs via endocytosis of the BoNTs-receptor complex inside the neurons; (iii) translocation of BoNTs L-chain from endocytosed vesicle to the neuronal cytosol; and, finally, (iv) zinc-endopeptidase activity on cellular targets [13,14,25]. Intracellular targets of BoNTs are three proteins involved in neuroexocytosis of the neurotransmitter synaptic vesicles. These proteins are: SNAP-25 (synaptosomal associated protein of 25 kDa), VAMP (vesicle associated membrane protein), also called synaptobrevin, and sintaxin. All these proteins are involved into assembly of the SNARE (soluble N-ethylmaleimide-sensitive factor attachment protein receptors) protein core complex, which is fundamental for correct docking and fusion of neurotransmitter vesicles with neuronal membranes [13,14,15,16,17,18,19,20]. The peculiar characteristic of BoNTs resides in their high affinity for one of the three SNARE proteins: SNAP-25 is cleaved by BoNT/A (the serotype most commonly used in clinical practice), /E and /C; VAMP/sinaptobrevin is the target of BoNT/B (another serotype used in clinic), /D, /F and /G; and sintaxin is cleaved only by BoNT/C. The cleavage of one of these proteins is sufficient to prevent the correct assembly of the SNARE core complex and the consequent fusion of synaptic vesicles with the neuronal presynaptic membrane, thus inhibiting the neurotransmitter release [19,20]. This effect is reversible, and the duration of action is dependent on the serotype. Further details on binding, internalization and mode of action of the different BoNTs serotypes can be found in other reviews [21,22,23,24,25,26]. 

## 3. Beyond Muscular Effects: Involvement of Molecules Modulating Pain

Studies on the pathophysiology of botulism revealed that neuromuscular paralysis is due to selective inhibition of evoked ACh release from cholinergic nerve endings at the skeletal neuromuscular junction [1]. This canonical effect of BoNTs gave the opportunity to use them as therapeutic agents in a variety of neurological disorders due to hyperfunctionality of cholinergic terminals [8]. However, BoNTs cannot be considered exclusively as ‘cholinergic’ toxins. Though they act preferentially on nerve terminals between motoneurons and muscle fibers, BoNTs can also block the neural transmission at other peripheral synapses, cholinergic or not [9]. This has indirectly been proved also by symptoms observed during botulinal neuromuscular paralysis, where autonomic nervous system dysfunction (parasympathetic, *i.e.*, cholinergic, and sympathetic, *i.e.*, adrenergic and noradrenergic) coexists with neuro-motor alterations [4,13]. 

Extensive *in vitro* and *in vivo* studies demonstrated that BoNTs (mainly BoNT/A) are able to block the Ca^2+^-evoked release of neurotransmitters, other than ACh [27]. As for examples, *in vitro* studies demonstrated that BoNTs inhibit various neurotransmitters’ release, including glutamate, GABA, aspartate, catecholamine, and noradrenaline, from cerebral synaptosomes, chromaffin cells, central neurons and hippocampal slice cultures [28,29,30,31,32,33,34,35,36,37,38]. *In vivo* studies, mainly using microdialysis techniques, demonstrated that BoNTs block both dopamine and monoamine release under various conditions [39,40,41,42]. Moreover, BoNTs block glutamate release also from non-neuronal cells, such as astrocytes [43,44,45]. Finally, there are many evidences in literature that BoNTs are able to block the release not only of classical neurotransmitters but also of neuropetides, such as Substance P (SP) [46,47,48,49] and calcitonin gene-related peptide (CGRP) [50,51,52,53,54,55]; neuropeptides whose role in pain modulation is known. These latter findings gave strong input in searches for alternative therapy against trigeminal neuralgia and various types of headache and migraine—neurological diseases where SP and CGRP release often act as a trigger point to exacerbate disease attacks. In these pathologies, BoNT/A probably exerts analgesia by blocking the neuropeptides’ release at the nociceptive nerve endings. Some encouraging results have been obtained in humans [56,57]. The effect of BoNT/A in tension-type headache, chronic daily headache, and/or migraine is not further considered in the present review. 

BoNT/A is effective in relieving pain in spastic and non-spastic muscle conditions in humans. This analgesic effect has generally been attributed to muscular relaxation. Notwithstanding, there are reports in the literature stating that patients experience pain relief shortly after BoNT/A treatment [58,59], *i.e.*, before of any muscle-relaxing action of the toxin, or that the pain relief is still maintained after muscle power returned to normality [60]. These results suggest that the analgesia attributed to BoNT/A may be due to more complex mechanisms than the simple muscular relaxation. In such cases, the pain relief cannot be ascribed to abolition of those factors, such as muscle tone, excessive muscle contraction and spasms, which are due to muscle hyperactivity [61]. In the next sections, we will discuss the evidences from animal models in favor of an analgesic action of BoNTs, with particular emphasis on pain not directly related to abnormal muscle hyperactivity, such as inflammatory and neuropathic pain. Animal model studies have been mainly performed with BoNT/A and /B and have been conducted by using the 150 kDa purified toxin or equivalent commercial products. 

In the following, we will indicate BoNT/A, or /B, indifferently from which preparation has been actually utilized.

## 4. Animal Models Predicting the Therapeutic Use of BoNTs against Pain

Generally, pain is divided into nociceptive and pathological pain. Nociceptive pain is caused by the sustained activation of peripheral nociceptors in response to peripheral tissue injury and can be classified as somatic or visceral pain according to where the pain arises. The extent of nociceptive pain normally reflects the extent of tissue damage and it disappears when the origin of nociceptive stimuli disappears. On the other hand, pathological pain often evolves toward a chronic condition in which pain exceeds the extent of tissue damage. Independently from the organs or tissues involved, chronic pain may be a consequence of either inflammatory processes (for example, rheumatoid arthritis in humans) and/or injury to the nervous system, both peripheral (PNS) (for example, postherpetic neuralgia) and central (CNS) (for example, spinal cord injury) nervous system, which is commonly known as neuropathic pain. A common feature of pain-related mechanisms is the activation of sensitization processes, plastic changes in PNS and CNS (adaptative or pathological) that lead to an enhanced response and/or decreased threshold to nociceptive stimuli. For example, under neuropathic pain conditions, both hyperalgesia (an exaggerated pain perception in response to a painful stimulus) and allodynia (the perception as painful of a stimulus that does not usually provoke pain) are commonly developed. In basic science, many animal pain models have been developed in order to mimic human pain conditions. In the next paragraphs of this section, an overview of the studies involving the use of BoNTs in animal pain models will be presented and relevant results discussed. 

### 4.1. Peripheral Inflammatory Pain Model

A direct involvement of BoNTs in mechanisms involved in pain modulation was first described by Cui *et al*. [62], who analyzed the effects of BoNT/A in formalin-induced inflammation as an animal model of inflammatory pain. Formalin-induced inflammatory pain model is extensively used for the evaluation of possible analgesic effects of test compounds. It is performed by injecting a diluted formalin solution into one hindpaw of rats, or mice, and by recording the formalin-evoked behaviors. The most characteristic behavioral response of rodents to formalin injection is the licking of the injected paw. This response appears biphasic, with an early phase of extensive licking due to peripheral sensitization of nociceptors, followed by an interphase with almost absence of licking activity due to activation of inhibitory descending pathways, and finally by a second phase of extensive licking, which essentially reflects the progression of peripheral inflammation together with the activation of central sensitization processes [63]. Cui *et al*. [62] found that a single peripheral subcutaneous injection of BoNT/A was able to reduce licking activity during the second phase, in a dose-dependent manner, with the absence of any obvious muscle weakness at doses below 30 U/kg. BoNT/A, administered at different time intervals prior to formalin testing, induced long-lasting analgesic effects, starting within the first 24 hours and lasting at least 12 days, the latest time point tested. The reduction of licking activity was accompanied by the inhibition of formalin-induced peripheral release of glutamate in the rat paw, and by the reduction of paw edema that is usually observed as a consequence of neurogenic inflammatory processes. This latter effect suggests that BoNT/A may reduce not only the release of glutamate, but also of SP and CGRP (not directly measured by the authors), involved in SP-mediated plasma extravasation and CGRP-mediated vasodilatation, respectively. Interestingly, BoNT/A did not reduce the initial phase of licking behavior and thermal sensitivity, indicating no effects of BoNT/A injection on acute pain. The authors postulated that BoNT/A is able to exert analgesic effects on formalin-induced inflammatory pain by inhibiting the peripheral stimulation-induced release of neurotransmitters, such as glutamate, and probably also of the neuropeptides SP and CGRP. Under this view, the analgesic activity of BoNT/A has been considered as a consequence of the inhibition of peripheral sensitization, resulting in indirect reduction of central sensitization processes [64,65].

Luvisetto *et al*. in mice [66] have essentially confirmed results of Cui *et al*. in rats [62]. These authors extended the analysis of BoNTs on formalin-induced inflammatory pain by considering for the first time the central (intracerebroventricular) *versus* peripheral (subcutaneous into hindpaw) effects. Moreover, they investigated two BoNTs serotypes, namely BoNT/A and BoNT/B. The authors demonstrated that pre-treatment (3 days) with BoNT/A is able to reduce the second phase of formalin independently from the route of administration, demonstrating that BoNT/A may act not only peripherally but also at the central level. On the contrary, BoNT/B did not reduce licking activity and, when centrally injected, had a hyperalgesic effect on the interphase of the formalin test. These results indicate a different mechanism of action of BoNTs, depending on the serotype and route of administration, and suggest that BoNT/A and BoNT/B are not interchangeable based on simple dose ratio, a point that is extremely important to take into account in a therapeutic perspective. Moreover, these observations were fundamental for the comprehension of the mechanism involved in the action of BoNTs: BoNT/A and BoNT/B may interact with inhibitory and/or excitatory systems in modulating persistent inflammatory pain. The different effects of the two serotypes may reflect differences in the expression of receptors and cellular targets of BoNT/A and BoNT/B (*i.e.*, SNAP-25 and VAMP/synaptobrevin, respectively), as well as in the expression of these targets into different neuronal populations.

A series of studies [67,68,69,70] showed that the vesicle recycling in hippocampal glutamatergic neurons is blocked by BoNT/A and BoNT/B while the same processes in hippocampal GABAergic neurons are blocked by BoNT/B but not by BoNT/A. Staining with various antibodies directed against SNAP-25 reveals the specific presence of this protein in glutamatergic neurons, while GABAergic neurons lack immunoreactivity for SNAP-25. In contrast, staining with antibodies directed against SNAP-23, a homolog of SNAP-25, shows its presence in both glutamatergic and GABAergic neurons. BoNT/A does not cleave SNAP-23 and this explains why BoNT/A is not able to block GABAergic neurons. On the other hand, BoNT/B acting on VAMP/synaptobrevin—another protein of the SNARE complex—is able to block GABAergic neurons. Considering all these pieces of evidence, the effect of BoNT/B on the interphase of the formalin test could be partially ascribed to a functional block of GABA inhibition on the primary afferent fibers in spinal dorsal horn. 

The study on the analgesic effects of BoNT/A on inflammatory pain was not restricted to formalin-induced pain and inflammation but was extended also to other models of inflammatory pain, such as that induced by carrageenan and capsaicin. Peripheral application of carrageenan or capsaicin produces hypersensitivity to thermal and mechanical stimuli as a consequence of inflammation due to injected chemicals irritants. Carrageenan promotes inflammation by activating proinflammatory cells and the resulting inflammatory edema is more extensive than edema induced by formalin. It causes hyperalgesia by promoting the peripheral release of mediators such as SP, glutamate, prostaglandins, histamine and serotonin, as well as release of glutamate, aspartate, SP, CGRP, nitric oxide, and prostaglandin E2 (PGE2) in the dorsal horn of lumbar spinal cord. On the other hand, capsaicin excites sensory neurons directly by acting on vanilloid receptors type 1 (VR-1), mostly expressed on C-fibers. VR-1 receptors are present on nerve fibers containing SP and CGRP that are released from nerve terminals after capsaicin application. Bach-Rojecky and Lacković [71] found that pre-treatment (6 days) with BoNT/A significantly reduced or completely abolished the enhanced sensitivity to mechanical and thermal stimuli provoked by peripheral carrageenan or capsaicin injections in rats, demonstrating the efficacy of peripheral BoNT/A pretreatment on the pain component of inflammatory process in experimental animals. In contrast with Cui *et al*. [62], BoNT/A had no effect on the carrageenan-induced paw edema. Following this last observation, in another study, Back-Rojecky *et al*. [72] analyzed if the analgesic effect of BoNT/A on carrageenan- and capsaicin-inflammatory pain was directly due to the anti-nociceptive effect or indirectly due to the anti-inflammatory effect. The authors confirmed that pre-treatment with the same dose of BoNT/A effective in reducing pain [71], had no effect on the size of carrageenan-induced paw edema, measured as paw volume and weight, or on the capsaicin-induced plasma extravasations (PE), measured by Evans blue as a marker of protein leakage. From this finding, they concluded that BoNT/A does not have a significant anti-inflammatory effect on the inflammation induced by capsaicin and carrageenan, indicating a possible dissociation between the effect of BoNT/A on pain and on inflammation: only the former was affected by BoNT/A through the inhibition of the release of neurotransmitters from the peripheral endings of sensory nerves. Similar results were obtained also by Favre-Guilmard [73], who reported that BoNT/A was not able to reduce carrageenan edema, indicating that the antihyperalgesic effects of BoNT/A are independent of an anti-inflammatory activity related to increased vascular permeability and adherence/infiltration of inflammatory cells. On the contrary, Carmichael *et al*. [74] evidenced contrasting results on the anti-inflammatory effects of BoNT/A on neurogenic inflammation. These authors found that peripheral subcutaneous application of BoNT/A reduced vasodilatation and PE evoked by saphenous nerve stimulation or topical administration of capsaicin in the rat hindpaw skin, while it had no effect on SP-induced PE or CGRP-induced vasodilatation. 

Although some controversies appear to be still unsolved, studies presented in this paragraph are clearly in favor of BoNT/A as a powerful drug against inflammatory pain and future research is necessary to better elucidate the mechanisms involved. 

### 4.2. Visceral Pain Model

The effect of BoNTs on visceral pain models has not been extensively studied. However, the efficacy of BoNTs on relieving visceral pain has been analyzed in models mimicking pain symptoms due to lower urinary tract disorder in humans, such as that due to bladder hyperactivity and prostatitis. It was observed that intravesicular administration of BoNT/A produced analgesia against acetic acid-induced bladder pain in rats by inhibiting the CGRP release from afferent nerve terminals [75]. In another model, namely the cyclophosphamide-induced cystitis in the bladder of rats [76], BoNT/A inhibited the cyclooxygenase 2 (COX-2) and the prostaglandin EP(4) receptor expression and suppressed bladder hyperactivity. Moreover, in a model of prostatitis, obtained by intraprostatic capsaicin injection in rats, BoNT/A inhibited COX-2 expression and suppressed prostatic pain [77]. These studies in animal models (see also [54]) have paved the way toward an extensive therapeutic application of BoNT/A in clinical treatment of human disorder related to lower urinary tract disorder [78].

### 4.3. Neuropathic Pain Model

Neuropathic pain is a kind of chronic pain resulting from injury to the peripheral or central nervous system. Common examples of neuropathic pain include postherpetic neuralgia, diabetic neuropathy, complex regional pain syndrome, and pain associated with spinal cord injuries. Evidence for utility of BoNT/A in relieving neuropathic pain symptoms in humans was first reported by Klein [79], who demonstrated the effectiveness of BoNT/A in four clinical cases in alleviating symptoms of different neuropathic pain, such as relapsing-remitting multiple sclerosis, postherpetic neuralgia, peripheral neuropathy, and severe tingling caused by herniation of cervical vertebrae at the level of C8. Since this initial observation, the action of BoNT/A in neuropathic pain has been also the focus of experimental studies with the aim to better define its role as an analgesic and to investigate the neural mechanisms involved.

Many models of neuropathic pain are currently in use both in rats and mice to mimic human peripheral neuropathic conditions [80]. Depending on the site of injury, the animal models of neuropathic pain are generally subdivided in central pain models (mainly based on spinal cord injury), peripheral nerve injury models (mainly injuries at the level of sciatic or spinal nerves), and peripheral neuropathy induced by diseases (postherpetic neuralgia and diabetic neuropathic pain models, chemotherapy-induced peripheral neuropathy models) [81,82,83,84,85,86]. Thermal and mechanical hyperalgesia as well as allodynia are hallmark properties of the experimental neuropathies.

The effect of BoNT/A on neuropathic pain was first analyzed on the peripheral neuropathy induced by partial sciatic nerve transection in rats [87]. In this study, a single peripheral injection of BoNT/A was sufficient to reduce both thermal and mechanical hyperalgesia without any apparent muscle weakness. The effect of BoNT/A became evident five days after toxin application and lasted for more than 10 days. In accordance with previous observations in other pain models [62,66], the authors found that BoNT/A had no effects on thermal and mechanical thresholds of sham operated rats, confirming that BoNT/A *per se* does not change nociception.

A second neuropathic pain model, used to test the efficacy of BoNT/A as an analgesic, was the L5/L6 spinal nerve ligation (SNL). In this study, Park *et al*. [88] considered allodynia rather than hyperalgesia as a behavioral parameter to assess the peripheral neuropathy. The authors found that a single peripheral administration (into the plantar skin of one hindpaw) of BoNT/A, dose-dependently reduced both mechanical and cold allodynia in SNL neuropathic rats. 

**Figure 1:**
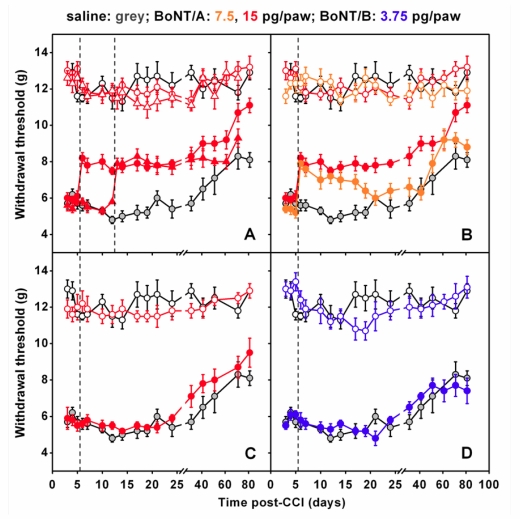
BoNT/A counteracts mechanical allodynia induced by chronic constriction injury (CCI) in mice. (**A**) Mechanical allodynia, expressed as withdrawal thresholds of applied force, of ipsi- and contralateral hindpaws as function of postoperative days in BoNT/A mice that received ipsi- and contralateral intraplantar injections of BoNT/A (15 pg/paw), either 5 (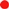, ipsi; 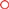, contra) or 12 (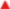, ipsi; 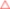, contra) days after CCI. Contralateral hindpaw was injected to check for possible neuroparalytic effect of peripheral BoNT/A injection. (**B**) Withdrawal thresholds of ipsi- and contralateral hindpaws as function of postoperative days after a intraplantar injections, into ipsi- and contralateral hindpaws, of BoNT/A 7.5 pg/paw (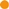, ipsi; 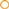, contra) or BoNT/A 15 pg/paw (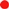, ipsi; 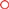, contra) at day 5 after CCI (dashed). (**C**) Effect of a single intraplantar injection of BoNT/A (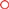, 15 pg/paw) into the hindpaw contralateral to the injury. Corresponding ipsilateral hindpaws were injected with saline (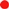). Injections performed at day 5 (dashed). (**D**) Withdrawal thresholds of ipsi- and contralateral hindpaws as function of postoperative days after a intraplantar injections, into ipsi- and contralateral hindpaws, of BoNT/B 3.75 pg/paw (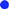, ipsi; 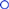, contra) at day 5 after CCI (dashed). For (A-D), control curves of ipsi- (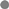) and contralateral (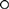) withdrawal thresholds in saline-injected hindpaws at day 5 are reported for comparisons. Dashed lines represents the day of injection while day 0 indicates the day of injury. For further details, see [89,91].

**Figure 2:**
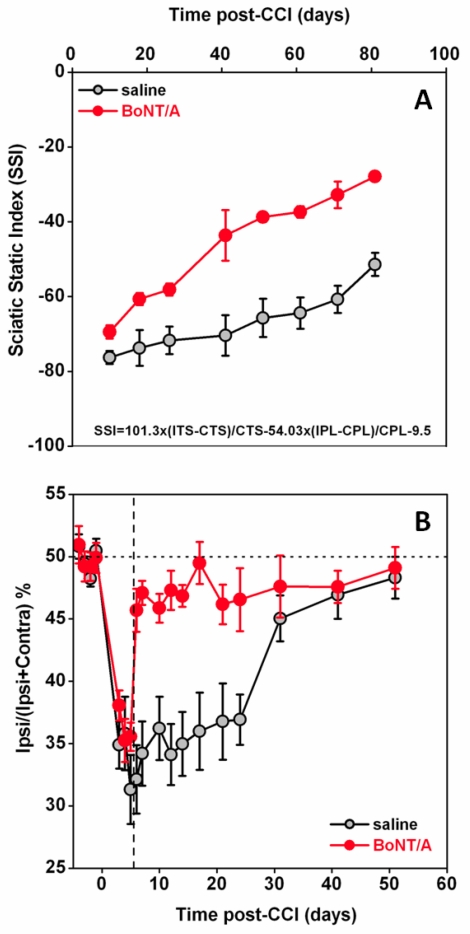
BoNT/A improves the functional recovery in mice subjected to CCI. (**A**) Effect of intraplantar injection of saline (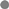) or 15 pg/paw BoNT/A (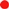) on the sciatic static index (SSI), calculated using the equation published in [126]. The hindpaw parameters to calculate SSI were obtained by walking track analysis and two footprint parameters were considered: the 1st–5th toe spread (TS) and the distance between the tip of the third toe and the most posterior aspect of the paw (PL). These variables were measured from at least five footprints, recorded on three different walking track runways, and entered into the sciatic static index-formula: SSI = +101.3 × (ITS-CTS) / CTS − 54.03 × (IPL-CPL) / CPL − 9.5. ITS: ipsilateral toe spread; CTS: contralateral toe spread; IPL: ipsilateral paw length; CPL: contralateral paw length. For SSI, a value of zero represents normal function and −100 represents complete loss of function of the sciatic nerve. (**B**) Effect of intraplantar injection of saline (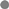) or BoNT/A 15 pg/paw (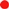) on weight bearing. Dashed line indicates the day of injection. Weight bearing was measured as percentage distribution of weight on the ipsilateral hindpaw. Ipsi- and contralateral weight distributions were measured using an incapacitance tester as described in [91].

Finally, a third animal model of peripheral neuropathy, the chronic constriction injury (CCI) of the sciatic nerve, was considered in some recent studies [89,90,91]. In these studies, authors found that a single injection of BoNT/A into plantar surface of mice injured paws, performed at five or 12 days after CCI, markedly antagonized mechanical allodynia induced by CCI [Figure 1(A)]. This effect was already evident 24 hours after BoNT/A injection, lasted for at least three weeks, and was dose-dependent [Figure 1(B)]. Interestingly, differently from BoNT/A treatment post surgery, if the BoNT/A injection preceded (3 days) the CCI, the antiallodynic effect was absent. These later findings underline the difference to what is observed in inflammatory pain models where BoNT/A is able to inhibit the occurrence of inflammatory process when injected prior to painful stimulus [62,66,71,72]. BoNT/A was able to reduce neuropathic symptoms only after neuropathy was already established but was unable to preventively protect against the onset of neuropathy. If BoNT/A was injected contralaterally to the lesion, anti-allodynic effects were not observed [Figure 1(C)]—an important point demonstrating the absence of a systemic diffusion in these experimental conditions. By using the same neuropathic pain model, authors observed that peripheral administration of BoNT/B did not exert antiallodynic effects [90] [Figure 1(D)]. This is in agreement with previous research of the same authors showing different effects of the two serotypes in inflammatory pain models [66]. It should be remembered that usable doses of BoNT/B are lower than those of BoNT/A since the serotype B shows a higher toxicity, thus limiting its use [66,90]. Furthermore, Marinelli *et al*. [91] observed that BoNT/A significantly improved the functional recovery of the injured paw, as assessed in mice by the sciatic static index of functional recovery, examined through the footprint walking tracks of both ipsi- and contralateral paws [Figure 2(A)], and by the weight-bearing incapacitance test [Figure 2(B)]. 

In addition to behavioral effects, some interesting data are emerging about the ability of BoNT/A to interfere with regenerative processes after nerve injury. It is known that the capacity of regenerating tissue is present in the peripheral nervous system [92]. When neuropathy is induced in animals, a composite process occurs that, together with axonal degeneration, is associated with infiltration of cells of the immune system such as macrophages and glial cells [93]. Schwann cells (SC) also have an important role in both degenerative and regenerative processes [94] and their interaction with macrophages is a determinant to provide a favorable microenvironment to axonal sprouting, elongation and maturation [95]. Marked changes in different gene and protein expression in sensory neurons are associated with nerve injury and the consequent process related to nerve regeneration. Marinelli *et al*. [91] have observed a significantly higher level of Cdc2, a prototypical cyclin-dependent kinase that regulates the mitotic phase of the cell cycle [96] and the cell migration processes [97], in nerve samples from neuropathic mice treated with BoNT/A with respect to samples from saline-injected mice. A recent paper from Han and colleagues [98] provides insight into the mechanisms of nerve regeneration, showing a new function of Cdc2 and other Cdk family members in the nervous system. They demonstrated that following sciatic nerve injury, isolated SC show elevated Cdc2 expression and enhanced migration and that inhibition of Cdc2 can block this effect, whereas increased Cdc2 expression enhances cell migration. As previously reported, SC play a role in injured nerves: they dedifferentiate to immature SC, acquire again the expression of molecules characteristic of embryonic development and up-regulate cytoskeleton constituents such as glial fibrillary acidic protein (GFAP) [99]. They regain capacity to proliferate, and their migration facilitates peripheral nerve regeneration after injury. A gradually enhanced expression of SC markers such as S100β and GFAP, proteins identifying myelinating and non-myelinating SC, respectively, also expressed in dedifferentiated cells, is observed after sciatic nerve injury [100,101,102]. This increase post-CCI is due to proliferative state of SC after injury. Marinelli *et al*. [91] demonstrated a role of BoNT/A in the modulation of these regenerative processes and their relationship with SC, showing a further enhanced expression of these proteins after *botulinum* treatment. 

Mika *et al*. [103] have added insights in the mechanism of action of BoNT/A, investigating molecular changes occurring in DRG and spinal cord after CCI to the sciatic nerve in rats. They observed that in the ipsilateral lumbar spinal cord of neuropathic rats, SNAP-25, prodynorphin and microglial (C1q) marker mRNAs were upregulated, while no changes occurred in neuronal (NOS1) and inducible (NOS2) nitric oxide synthase, GFAP, proenkephalin and pronociceptin mRNAs. In the DRG, ipsilateral upregulation of prodynorphin, pronociceptin, NOS1, NOS2, C1q and GFAP mRNAs, downregulation of proenkephalin and no changes in SNAP-25 mRNA were observed. A single intraplantar BoNT/A (75 pg/paw) injection induced long-lasting antinociception in the CCI model. BoNT/A diminished injury-induced ipsilateral spinal upregulation of SNAP-25 and C1q mRNAs and, in the ipsilateral DRG, reduced SNAP-25 and upregulated prodynorphin, pronociceptin and NOS1 mRNAs, with a significant decrease of C1q-positive cell activation. These evidences demonstrate that peripheral administration of BoNT/A attenuates neuropathic pain-related behavior by modulating several proteins in DRG and spinal cord, structures distant from the peripheral injection site. Silenced microglia/macrophages after BoNT/A administration could be secondary to the inhibition of neuronal activity, and such a decrease of neuroimmune interactions could be the key for a long-lasting BoNT/A effect in neuropathic pain. 

Trigeminal neuralgia is a neuropathic pain disorder characterized by recurrent episodes of intense, lancinating pain felt in one or more divisions of the trigeminal distribution, whose onset may be spontaneous or due to stimulation of a trigger point on the face or in the oral cavity. An animal model of such pathology is the unilateral infraorbital nerve constriction (IoNC) in rats [104]. IoNC produces long-lasting neuropathy behavior, characterized by the head withdrawal to mechanical stimulation in the whiskers pad area, concomitant with faster onset and increased magnitude of transmitter release from somata of trigeminal ganglion (TRG). By using the IoNC pain model, Kitamura *et al*. [105] found that peripheral injection of BoNT/A, performed three days postinjury, alleviated the IoNC-induced neuropathy behaviors and decreased the exaggerated neurotransmitter release in neurons acutely isolated from TRG ipsilateral to IoNC; the anti-allodynic effect being maintained for at least two weeks. 

In another study, Favre-Guilmard *et al*. [73] compared different commercial preparations of BoNT/A on mechanical hyperalgesia induced by paclitaxel in rats. This model mimics the induction of peripheral polineuropathy that follows chemotherapy, which represents a significant limiting problem in clinical therapy. In this research, the reliable bilateral mechanical hyperalgesia induced by repeated injections of paclitaxel was affected by the injection of BoNT/A that produced a significant antihyperalgesic effect in the injected paw of neuropathic animals three days after administration. The effects of BoNT/A was also tested in an experimental diabetic neuropathy model in rats [106]. In this study, rats were made diabetic by a single intraperitoneal injection of streptozotocin, and developed hyperalgesic behavior in sensitivity to mechanical, thermal and chemical noxious stimuli; BoNT/A was effective in reducing hyperalgesia at day 5 after the peripheral toxin injection and 24 hours after intrathecal injection [107].

### 4.4. Other Pain Models

BoNT/A has demonstrated efficacy in relieving pain symptoms also of inflammatory arthritic pain [108]. Two murine models were considered: (i) the acute inflammatory arthritis produced by intra-articular injection of carrageenan and, (ii) the chronic inflammatory arthritis by intra-articular injection of Freund's complete adjuvant (CFA) [109]. In these models, pain relief was assessed by tenderness (evoked pain by touching the affected area) measures and correlated to spontaneous nocturnal wheel-running. Narcotic analgesics were effective in both models, but in fully analgesic doses they impaired wheel-running activity. Intra-articular injection of BoNT/A significantly reduced arthritis joint tenderness, both in acute and chronic inflammatory arthritis, and normalized impaired spontaneous wheel running in mice with chronic inflammatory arthritis but not in those with acute inflammatory arthritis. These results suggest that intra-articular injection of BoNT/A is a promising therapy for chronic inflammatory arthritis but may not be effective for acute arthritis pain.

The post-surgical pain is a type of intense pain affecting nearly 50% of patients subjected to surgical operations. As with other kinds of pain, also post-surgical pain is treated with opioid and non-opioid drugs, but it often persists and gives rise to primary and secondary hyperalgesia. The most common experimental approach to study postsurgical hyperalgesia is the incisional model of pain [110]. Diverse drugs reduce incision-induced mechanical hyperalgesia in rats, but only morphine has been proven to be 100% effective, however beneficial effects last only for a few hours. Filipovic *et al*. [111] reported that a single subcutaneous injection of BoNT/A into plantar surface of the injured hindpaw, completely abolished secondary hyperalgesia after gastrocnemius incision in rats. What is more interesting, is that a single injection was enough to induce antihyperalgesic effects and that these effects lasted for at least 10 days starting from day 5 after injection. 

## 5. BoNT/A-Induced Analgesia: A Closer Look

All the evidence in favor of analgesic properties of BoNT/A in a wide variety of pain models pose some interesting questions about the mechanism of action. In the recent years, two main points emerged about the effects of *botulinum* neurotoxins: the first one is that these molecules are effective not only in peripheral but also in the central nervous system. The second important point is that part of the effects induced by BoNT/A administration are observable distant from the site of injection. 

Under therapeutic treatment of muscle hyperactivity conditions, BoNT/A is locally applied, with little or absent diffusion, and its paralyzing action remains confined to the nerve-muscle junction, close to the injection site. This assumption constituted a “dogma” for many years of the use of these neurotoxins in human therapy. Some experimental evidence obtained in recent years from basic scientific research challenge this dogma and raise concern that while most of the effects are localized close to the injection site, BoNT/A can also act at distant sites. Actually, the possibility that BoNT/A could reach the CNS by retrograde transport was already suggested many years ago by experiments with radiolabeled BoNT/A [112,113]; however, it was observed that the retrograde axonal transport was so slow that the toxin was likely to be inactivated before it reached the cell soma [114].

More recent studies support the retrograde transport of BoNT/A. In particular, Antonucci *et al*. [115] demonstrated that BoNT/A may be retrogradely transported by central neurons and motoneurons and then transcytosed to afferent synapses. In their very elegant work, the authors presented three pieces of evidence in favor of axonal migration of BoNT/A. First, they showed that after a unilateral intrahippocampal injection of BoNT/A, cleaved SNAP-25 was detectable also in the contralateral untreated hemisphere. Second, after injection of BoNT/A into the optic tectum, cleaved SNAP-25 appeared also in synaptic terminals within the retina. Since the natural target of BoNT/A is the neuromuscular junction, in a third experiment, authors chose to test the spread of toxin in the facial motoneurons projecting to the whisker muscles. After BoNT/A injection at the center of the whisker pad, cleaved SNAP-25 was detected in the facial nucleus, confirming a possible migration of toxin also along motoneurons. Summarizing, after BoNT/A injection into central and peripheral regions, truncated SNAP-25—the target protein of SNARE complex selectively cleaved by BoNT/A—appeared not only at the injection site but also in distant regions projecting to the infusion area. The retrograde spread was blocked by the microtubule depolymerizing agent colchicine, pointing to an involvement of microtubule-dependent axonal transport. In another research, when BoNT/A was injected in the whisker pad area, Kitamura *et al*. [105] observed a strong inhibition of the increased KCl-evoked neurotransmitter release from trigeminal ganglion neurons after infraorbital nerve constriction.

Similarly to Antonucci *et al*. [115], the results reported in another study [116] may not be completely accounted for without considering a possible axonal transport of the toxin distant from the injection site. Bach-Rojecky and Lackovic [116] used the model of unilateral injection of acidic saline solution into gastrocnemius muscle as an animal model of muscle hyperalgesia. In this model, both ipsi and contralateral hyperalgesia are detectable, with contralateral hyperalgesia being considered as centrally mediated secondary hyperalgesia. BoNT/A, subcutaneously injected into ipsilateral hindpaw, exerted antihyperalgesia both on ipsi- and contralateral hindpaws. The effect on both sides was evident on day 5 and was of similar intensity. Interestingly, when colchicine was injected into ipsilateral sciatic nerve, one day before the ipsilateral BoNT/A injection, the antihyperalgsic effect of BoNT/A on both sides was not observed. On the contrary, if colchicine was injected into contralateral side, opposite to the site of pain induction and BoNT/A injection, it did not prevent the antihyperalgesic effects of BoNT/A on both sides. Finally, when BoNT/A was applied intrathecally, the bilateral hyperalgesia was also reduced. Altogether, these results cannot be explained without the involvement of the central nervous system and without the assumption that BoNT/A is transported from the site of injection. Bilateral effects after unilateral BoNT/A injection were also observed in paclitaxel-induced neuropathy in rats [73], and in streptozotocin induced diabetic neuropathy in rats [107]. 

Even if an easy generalization of the results derived from animal studies to humans is not recommended and a number of factors (anatomical differences, doses, volume of dilution, *etc.*) have to be taken into account (see [117]), these studies represent a crucial step in the comprehension of *botulinum* neurotoxin transport mechanism.

## 6. Proposed Mechanism of Action for BoNT/A Analgesia

**Figure 3:**
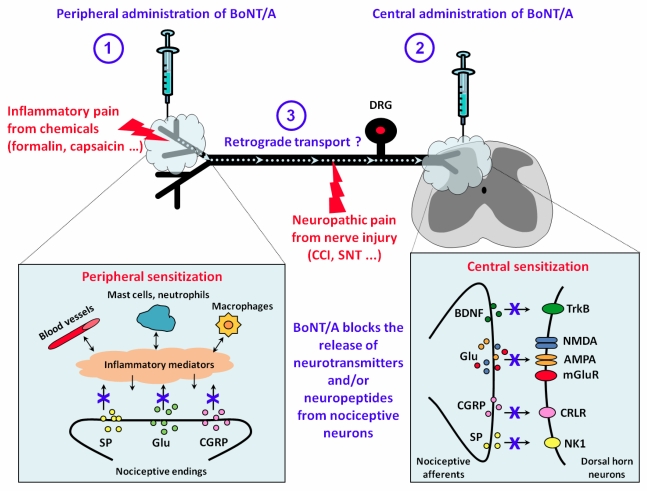
Proposed mechanism of action of BoNT/A in pain modulation. In this scheme, the analgesic effects of BoNT/A is thought to be exerted through the inhibition of the release of neurotrasmitters and/or neuropeptides from nociceptive neurons both at peripheral and/or central level, depending on the route of administration. (1) The peripheral analgesic effect of BoNT/A, as observed after subcutaneous or intraplantar injection, may be a direct consequence of the reduced release of neuromodulators from nociceptive endings. By inhibiting this release, peripherally injected BoNT/A may reduce directly the peripheral sensitization. (2) When intrathecally injected, BoNT/A may inhibit the release of neurotransmitters and/or neuropeptides from central terminals of nociceptive afferents reducing the central sensitization. (3) Central analgesic effects may be also induced by retrograde transport along axons of peripherally injected BoNT/A. These peripheral and central effects may occur, and partially overlap between them, both in inflammatory and neuropathic pain models. Symbols: AMPA, 2-amino-3-(5-methyl-3-oxo-1,2-oxazol-4-yl) propanoic acid receptors of glutamate; BDNF, brain derived neutrophic factor; CCI, chronic constriction injury; CGRP, calcitonin gene related protein; CRLR, calcitonin-receptor like receptor; DRG, dorsal root ganglions; Glu, glutamate; mGluR, metabotropic glutamate receptors; NK1, neurokinin 1 receptors; NMDA, N-methyl-D-Aspartate receptors of gluatamate; SNT, spinal nerve transection; SP, substance P; TrkB, tyrosin kinase B receptors.

Considering all the results reviewed, we suggest a possible model of action of BoNT/A on pain transmission, as in the scheme depicted in Figure 3. In this scheme, two pain conditions are considered: inflammatory, such as that induced by chemicals (formalin, capsaicin, *etc.*) or neuropathic pain derived from nerve injury (CCI and spinal nerve ligation or transection). BoNT/A may be administered peripherally by subcutaneous or intraplantar injections, or centrally by intrathecal or intracerebroventricular injections. Depending on the site of injection, BoNT/A may differently exert its effects. By inhibiting the release of neurotransmitter and/or neuropeptides from nociceptive endings, peripherally injected BoNT/A may reduce directly the peripheral sensitization and indirectly the central sensitization, (Figure 3, point 1). In the same way, intrathecally injected BoNT/A may inhibit the release of neurotransmitters and/or neuropeptides from central terminals of nociceptive afferents, reducing the central sensitization (Figure 3, point 2). Finally, peripherally injected BoNT/A may be retrogradely transported along axons of peripheral nerves, thus acting also on central sensitization processes (Figure 3, point 3). This retrograde transport may be responsible for the displacement of peripheral injected BoNT/A at the level of the spinal dorsal horn where it exerts a possible inhibition of spinal release of neurotransmitters. Moreover, in a neuropathic pain model, the retrograde transport of BoNT/A, after being peripherally injected at the level of the paw, may be involved in the observed regenerative processes at the level of injured nerve [91] and the inhibitory effects at level of DRG and spinal cord [103]. 

Whatever the mechanism, the analgesic effects observed, as well as the lack of deleterious side effects, imply that, while experimental research is going to continue, the potential use of BoNT/A for therapy is strongly supported.

## 7. Conclusions and Future Perspectives

In summary, BoNTs are zinc metalloendoproteases that exhibit extraordinary specificities for proteins involved in the neurotransmitter release process, whose toxicity makes them responsible for animal and human botulism. However, these extremely poisonous molecules can become useful therapeutic agents in a number of expanding applications in human medicine, including modulation and alleviation of pain conditions. This is particularly evident for one of the BoNTs serotypes, the serotype A. One of the reasons for this success is the evident advantage of using a drug with a prolonged duration of action, thus allowing a long interval between treatments, as demonstrated by all the studies reviewed in this present review. 

Another important aspect is that a single injection of BoNT/A is able to induce long-term analgesic effects in many different pain models, from inflammatory to neuropathic. The indications for BoNTs therapy will, no doubt, continue to expand and ongoing efforts to elucidate BoNTs’ mechanisms of action for reducing pain will provide an essential foundation for developing future therapeutic strategies. Although BoNTs may benefit pain syndromes and can theoretically be administered to treat many pain conditions, its use appears restricted to peripheral administration and the possible lethal consequences of systemic administration of the toxin limit its potential for clinical trials. It should be recognized that peripheral administration is also not completely devoid of risk and needs further investigations, with particular reference to the central action. Much evidence has been presented for retrograde transport of BoNT/A distant from the site of injection and more in depth studies will be necessary to characterize the mechanisms of this retrograde transport. Among others, as suggested by Caleo *et al*. [118], an important aspect that has to be better clarified is whether the central effects depend on the dose used as well as on density of innervations and levels of expression of toxin receptors.

In an attempt to bypass the risks due to toxicity, a desirable characteristic for future BoNTs products would be an increased specificity for pain conditions. This may be achieved by replacing the native binding domain with another protein to re-direct the light chain to a different nerve or cell [119]. Such modifications may enable BoNTs to treat pain without engendering weakness of nearby muscles. In this context, an innovative class of biopharmaceuticals obtained by recombinant techniques has been recently proposed [120,121,122,123,124,125]. These recombinant proteins, also named as “targeted secretion inhibitors”, incorporate the light chain fragment of BoNT/A expressing the endopeptidase activity fused with a protein that selectively binds to specific neuronal or non-neuronal cells. A promising example of these proteins is the recombinant protein obtained by fusion of the light chain of BoNT/A with lecitin from *Erythrina cristagalli,* which binds to galactose-containing carbohydrates selectively present on nociceptive afferents in the central and peripheral nervous system. This recombinant protein has been demonstrated to be effective in inhibiting the release of SP and glutamate from DRG neurons in culture [122] and to relieve pain symptoms in different pain models [123]. 

Since BoNT/A is actually under clinical trials for treatment of pain in various types of headache and migraine conditions [57], the translation of encouraging results from preclinical studies in inflammatory and neuropathic animal pain models, to clinical treatments of chronic pain in humans can be considered a crucial step for human health. However, more in depth researches are still necessary to better establish the exact mechanism responsible for analgesic effects of BoNT/A. This will improve our knowledge about this relevant thematic of research and ongoing efforts to elucidate BoNTs’ mechanisms of action for reducing pain will provide an essential foundation for developing future therapeutic strategies.
